# Pubertal induction in Turner syndrome without gonadal function: A possibility of earlier, lower-dose estrogen therapy

**DOI:** 10.3389/fendo.2023.1051695

**Published:** 2023-03-28

**Authors:** Yukihiro Hasegawa, Tomonobu Hasegawa, Mari Satoh, Kento Ikegawa, Tomoyo Itonaga, Marie Mitani-Konno, Masanobu Kawai

**Affiliations:** ^1^Division of Endocrinology and Metabolism, Tokyo Metropolitan Children’s Medical Center, Tokyo, Japan; ^2^Department of Pediatrics, Keio University School of Medicine, Tokyo, Japan; ^3^Department of Pediatrics, Toho University Omori Medical Center, Tokyo, Japan; ^4^Clinical Research Support Center, Tokyo Metropolitan Children’s Medical Center, Tokyo, Japan; ^5^Department of Pediatrics, Oita University Faculty of Medicine, Oita, Japan; ^6^Department of Bone and Mineral Research, Research Institute, Osaka Women’s and Children’s Hospital, Osaka, Japan; ^7^Department of Gastroenterology, Nutrition, and Endocrinology, Osaka Women’s and Children’s Hospital, Osaka, Japan

**Keywords:** Turner syndrome, hypogonadism, estrogen, induction, puberty

## Abstract

Delayed and absent puberty and infertility in Turner syndrome (TS) are caused by primary hypogonadism. A majority of patients with TS who are followed at hospitals during childhood will not experience regular menstruation. In fact, almost all patients with TS need estrogen replacement therapy (ERT) before they are young adults. ERT in TS is administered empirically. However, some practical issues concerning puberty induction in TS require clarification, such as how early to start ERT. The present monograph aims to review current pubertal induction therapies for TS without endogenous estrogen production and suggests a new therapeutic approach using a transdermal estradiol patch that mimics incremental increases in circulating, physiological estradiol. Although evidence supporting this approach is still scarce, pubertal induction with earlier, lower-dose estrogen therapy more closely approximates endogenous estradiol secretion.

## Introduction

Turner syndrome (TS) is one of the most common chromosomal abnormalities and occurs in phenotypic females with one X chromosome and absence of all or part of the second X chromosome. TS is linked to a wide range of clinical features, including short stature and hypogonadism. Primary hypogonadism is an extremely common feature of this disorder. At outpatient clinics for children with TS, 80% or more of the patients require estrogen replacement therapy (ERT) (1). In this monograph, “induction” refers to the initial phase of ERT through puberty, from the initiation of estrogen therapy to the addition of progestin.

The type of ERT recently advocated worldwide for induction was originally published following the 2016 Cincinnati Meeting (2) and was endorsed by the European Society of Endocrinology, the Endocrine Society, the Pediatric Endocrine Society, the European Society for Pediatric Endocrinology, the European Society of Human Reproduction and Embryology, the American Academy of Pediatrics, and the Society of Endocrinology (United Kingdom) (3). In line with the consensus reached at the Cincinnati Meeting, a more precise, practical review of puberty and estrogen replacement was released in 2018 (3).

Several excellent guidelines were issued following the two previously mentioned publications (2, 3). Publications before 2016 were summarized in one of the Tables of reference 3. **Table 1** in the present monograph summarizes the relevant points of studies published in 2016 or later (2–14); some of these focused on using transdermal 17β estradiol (E2), while others were more practical in their recommendations. As seen in **Table 1**, most expert opinions and guidelines from around the world agree on the following points regarding induction therapy in TS without endogenous estrogen production:

- Therapy should be started at age 11–12 years.
- The dosage should gradually be increased, starting at 3 to 10% of the adult dosage.
- Progestin-induced menarche should be considered between two to three years after pubertal induction.
- Progestin may also be indicated to induce menstruation in patients who have irregular menses when receiving estrogen only.

**Table 1:** Guideline publications on pubertal induction in Turner syndrome.

Year	Ref.	Country or society		Age (years)	Estrogen preparation	Initial dose	Timing of progestin therapy	Progestin preparation	Comment
2022	(4)	USA (NIH)	Pediatric and Gynecology perspective	11~12	preferably E2-TD	1/32 of adult dose	breakthrough bleeding or 2 yrs after ERT	(oral therapy unless stated otherwise) MPA, micronized progesterone, levonorgestrel intrauterine device	Practical approach is presented
2021	(5)	Italy	Review	11~12	preferably E2-TD	10~25% of adult dose	2-3 yrs after ERT or mature uterus	MPA, micronized progesterone, or dydrogesterone	Maturity of the uterus is stressed as progestin initiation
2020	(6)	France	French National Protocol	11~12	preferably E2-TD	~1/10 of adult dose	breakthrough bleeding or 2 yrs after ERT	micronized progesterone, or dydrogesterone	Extensive guideline
2019	(7)	ESPE TS study group	Expert opinion	11~12	E2-TD	1/24~1/16 of adult dose	mature uterus and thick endometrium	MPA, micronized progesterone, or dydrogesterone	ESPE study group consensus
								(should not be given automatically after 2 yrss of ERT)	Maturity of the uterus is stressed as progestin initiation
2019	(8)	North American Society for Pediatric and Adolescent Gynecology							
			Review	11~12	E2-TD	3-7 mcg/d	breakthrough bleeding or 2~3 yrs after ERT	MPA, micronized progesterone, various types of estrogen and progestin combinations, in addition to oral contraceptions	Estrogen and progestin preparations are listed
					E2-oral EE2-oral CE-oral	0.25 mg/d 2 mcg/d ~0.15 mg/d			
2018	(3)	Practical recommendation following the 2017 guideline below				10~25% of adult dose	breakthrough bleeding or 2 yrs after ERT	MPA, micronized progesterone, various types of estrogen and progestin combinations, in addition to oral contraceptions	Estrogen and progestin preparations are listed
			Review	11~12	E2-TD E2-oral EE2-oral CE-oral	3-7 mcg/d 0.25 mg/d 2 mcg/d ~0.15 mg/d			
2017	(2)	The international Turner syndrome consensus group							Endorsed by 7 societies
			Expert opinions	11~12	preferably E2-TD	3-25% of adult dose	breakthrough bleeding or 2 yrs after ERT	MPA, micronized progesterone	Mostly accepted
2017	(9)	England	Expert opinions			10~25% of adult dose	breakthrough bleeding or 2.5 yrs after ERT	preferably Urogestan (vaginal, authentic progesterone), or MPA	Practical suggestions with three types of estrogens
					E2-TD (25 mg patch) E2-oral EE2-oral	1/4 patch for 3~4 days/wk 0.5 mg ALD 2 mg/d			
2017	(10)	Korea	Review (personal)	Not described	E2-oral	50-70 ng/kg/day	breakthrough bleeding or 2 yrs after ERT	Not described	Published in the official journal of KSPE
2016	(11)	Poland	Expert opinions	12~14	preferably E2-TD	1/10 of adult dose	at least 2 yrs after ERT	Not described	Published before ref. 2
2016	(12)	The European Society of Human Reproductive and Embryology		12			breakthrough bleeding or 2 yrs after ERT	micronized progesterone or dydrogesterone	Published before ref. 2
					E2-TD	1/32~1/16 of adult dose (6.25 mcg/day)			
					E2-oral	1/16~1/8 of adult dose (see comment)			(5 mcg/kg/day or 0.25 mg)
2009	(13)	Japan	Expert opinions (in Japanese)	12~14	CE	1/10 of adult dose	breakthrough bleeding or 2 yrs after ERT	MPA	Endorsed by JSPE
					E2-TD	1/9 of adult dose	breakthrough bleeding or 2 yrs after ERT	MPA	
2007	(14)	A guideline of the Turner syndrome study group		12~13			breakthrough bleeding or 2 yrs after ERT	micronized progesterone or dydrogesterone	Published before ref. 2 (cited a long time before ref 2)
		Expert opinions (cited a long time before ref 2)			E2-TD	1/32~1/16 of adult dose (6.25 mcg/day)			
					E2-oral	1/16~1/8 of adult dose (see commnet)		(5 mcg/kg/day or 0.25 mg)

A extensive summary of estrogen treatment of pubertal induction in TS (from 2001 to 2015) was published by Klein et al. in 2018.

We focused publications after 2015, with an exception of 2007, 2009 publications.

E2, 17β estradiol; EE2, ethinyl estradiol; CE, conjugated estrogen; MPA, medroxyprogesterone acetate; E2-TD, E2 transdermal; ERT, estrogen replacement therapy; ALD, alternate days.

#http://jspe.umin.jp/medical/files/turner.guideline.pdf; the text is in Japanese and is explained here.

The guidelines and reviews on TS covering pubertal induction have demonstrated variable methods of starting induction. E2 is most commonly recommended for induction, preferably *via* transdermal administration (2, 3, **Table 1**). In practice, many types of E2 patch are available in different parts of the worlds, as listed by Klein et al. (3). In addition, other types of estrogen, such as oral E2, synthetic ethinyl estradiol (EE2), and conjugated estrogen (CE) produced from horse urine, are also used worldwide (15-19 in **Table 2**). The list in **Table 2** is not exhaustive, but shows the methods used for inducing puberty in five institutions, regions, or countries after the release of the Cincinnati Meeting (2). The points outlined above can also be applied to oral E2, EE2, and CE as well as transdermal E2 application. A more extensive list of estrogen preparations was published by Klein et al. (3).

**Table 2:** Real-world estrogen induction in patients with Turner syndrome.

Year	Ref.	Country (survey)	N	Age (yrs) of ERT	Estrogen	Initial Dose	Years to menarche	Progestin	Comments
2022	(15)	Taiwan (national)	27	13.6 (12.4-15.6) median (IQR)	CE	25% of AD	2.7 (1.5-3.5) after ERT	MPA	E2-TD not available
2020	(16)	Arab region (regional)	106	84.2 % at 11 or older	29.7% EE2	ND	33.3% after bleeding	42.3% oral contraceptives	E2-TD not available
				5.3% at 4-7	16.6% CE		26.7% 2-3 yrs ERT	19.2% MPA	
2019	(17)	England (1 center)	624	14 (5 -23.4) median (5th-95th %tiles)	EE2	25% of AD	ND	ND	The largest number
2018	(18)	Poland (1 center)	49	15.1 (11.7-17.8) mean (SD)	E2-TD	12.5 mcg/day	1.82 yrs after ERT	ND	Late induction
2018	(19)	Turkey (national)	18 (center)	12-13 yrs in 13	11: E2-TD 5: oral estrogen 1: EE2	ND	61% after bleeding	ND	

The list is not all inclusive.

E2, 17β estradiol; EE2, ethinyl estradiol; CE, conjugated estrogen; MPA, medroxyprogesterone acetate

E2-TD, E2 transdermal.

ERT, estrogen replacement therapy.

ND, not described.

AD, adult dose.

In addition to reviewing the current understanding of pubertal induction in TS, the present monograph attempts to explain the rationale for the induction therapy and suggests an ideal method of induction in patients with TS without endogenous estrogen production. This method is not evidence-based but involves “earlier and lower-dose estrogen” than current, most frequently recommended forms of induction therapy.

## Gonadal function in Turner syndrome

### Pubertal development in healthy subjects and patients with TS

Between 1977 and 2013, the average, initial age of breast development decreased by almost three months per decade worldwide (20). The median age at Tanner Breast 2 (95% confidence interval [CI]) ranged from 9.8 to 10.8 years in Europe, 9.7 to 10.3 years in the Middle East, 8.9 to 11.5 years in Asia, 8.8 to 10.3 years in the United States, and 10.1 to 13.2 years in Africa (20). Even in patients with TS, spontaneous puberty reportedly began earlier than in the past, with numbers of such cases increasing significantly from pre-1980 to 2004 (21). The median age of menarche internationally was reportedly to be 12 to 13 years (22, 23).

Delayed or absent puberty is a common finding in TS. Menarche occurs in approximately 20% of TS patients seen in outpatient clinics worldwide (24–28). Aso et al. (24) demonstrated that, in Japan, only 5% of teenaged patients with TS who were followed up had regular, spontaneous menstruation at age 16 years. Thus, not all adult patients with TS with a history of spontaneous menarche can maintain their cycle.

Patients with TS who require pubertal induction therapy at a young age typically have no spontaneous breast development by 12 to 13 years. It is important to differentiate true thelarche from lipomastia, especially in overweight patients with TS. However, given that some patients with TS are unlikely to enter puberty or to have residual endogenous estrogen production indicated by an increased FSH level and/or streak gonads on imaging, pubertal induction therapy may be started earlier, for instance, around age 10 years, which is the average age of breast development in Europe and Asia (20, 23). On the other hand, in cases where it is unclear whether estrogen production will be absent or delayed, pubertal induction therapy should not be started until confirmation is obtained by physical examination and elevated FSH (> 40-50 mIU/mL depending on the assay used) and low estradiol (< 40-50 pg/mL) are recorded at least twice one month apart. Of course, delaying diagnosis until age 15 years, for example, in patients with no pubertal development, will inevitably entail a delay in induction as well. Furthermore, pubertal development in TS can be arrested at any stage during puberty, such as at breast development, menarche or later.

## Pubertal induction in TS without gonadal function

### Matters for patients and their caregivers to consider puberty induction

In pediatric medicine, the physician-patient-caregiver partnership is crucial. Physicians must understand the importance of this when treating patients with TS. Maintaining excellent rapport with patients is essential, as sensitive, psychosocial issues are often involved in patient care. Discussing concerns with patients and their guardians to support them holistically with other co-medicals is critical to managing hypogonadism in TS. Absent or delayed puberty may cause significant emotional and psychosocial morbidity [reviewed in ref. (29)]. Indeed, low self-esteem and social adjustment issues in young women with TS were reportedly influenced by the quality of pubertal management (30).

First, the patients and their parents should be made to understand healthy pubertal development. The possibility of delayed puberty should also be explained, especially in cases where it is likely to occur. Physicians should speak directly to their patients using understandable language and tailor their approach to each patient’s personality, intelligence, and developmental stage. The following information about induction treatment should be shared with the patients and their guardians:

- Estrogen replacement therapy is necessary in most patients with TS.
- One of the determinants of adult height is the timing of pubertal development, whether spontaneous or induced.
- An extreme delay in breast development may have unfavorable effects on psychosocial development and bone health.
- Various types of estrogen formulations and methods of administration are available, but transdermal administration of natural estrogen is currently recommended.

Medically speaking, the likelihood of spontaneous puberty must be considered before induction therapy. There are three markers capable of predicting pubertal outcomes in TS. The first, karyotype analysis, is a conventional method of predicting pubertal development. Patients with TS with 45,X/46,XX or 45,X/47,XXX, especially with a low mosaic ratio of 45,X, are more likely to be able to become pregnant naturally; thus, breast development and menarche are also likely to occur normally (31, 32). One limitation of karyotype analysis is that the mosaic ratio derived from blood cells, a common source, does not necessarily match that derived from other tissues, such as the ovaries (33, 34).

Second, the FSH level may serve as an index of spontaneous and cyclical menstruation in TS (24, 35, 36). Aso et al. investigated the utility of FSH level in 50 subjects aged 10–12 years with TS as an index of spontaneous and cyclical menstruation (24) and found that this occurred before age 16 in subjects with serum FSH <10 mIU/mL at age 10 to 12 years. The sensitivity and specificity of both was 90%. Similarly, Hankus et al. recommended FSH 6.7 mIU/mL as a criterion of menstruation onset in patients aged 6 to 10 years (35).

Third, Lunding et al. reported the use of the anti-Mullerian hormone (AMH) level as a predictor of pubertal development (37) and demonstrated that AMH < -2 SD during the prepubertal period predicted the failure of puberty onset. AMH was also able to predict imminent premature ovarian insufficiency (POI) in adolescent and adult patients with TS. No prepubertal patients with AMH < 4 pmol/L experienced spontaneous puberty, and adolescent patients with AMH < 5 pmol/L experienced imminent POI, consistent with the findings of several studies (38–41).

### Providing adequate estrogen and progestin in TS without gonadal function

#### Estrogen

Appropriate estrogen dosage is essential for pubertal induction. First, estrogen should be administered systemically because the ovaries normally release estrogen into the bloodstream. A practical method of administration in children is transdermal and uses a patch or gel (3). Transdermal patches are recommended by most of the current guidelines (2-14 in **Table 1**) because they are easy to use. However, patches are not yet available everywhere. Klein et al. recently extensively summarized current estrogen formulations (3). Administration of E2 or physiological estrogen allows the serum estrogen levels to be measured. In particular, the serum level achieved through transdermal administration ideally corresponds to the normal value per pubertal stage.

While transdermal administration of E2 has a number of the well-known, physiological advantages (**Table 3**), the oral administration of E2 has a rather salient disadvantage that is related to the first-pass effect. When taken orally, E2 is exposed to the liver through the portal vein after digestive absorption. However, there is little research directly comparing the transdermal and oral methods of administration during pubertal induction (42–45). Only one large study (42, 43) directly compared methods of pubertal induction with E2 in TS. **Table 3** shows more favorable, short-term outcomes for transdermal administration than for oral administration, but while the former may appear to be superior, its long-term effects are still unknown.

**Table 3:** Direct comparison of oral versus transdermal estrogen initiation in children with TS.

Year	Ref.	Oral preparation	TDpreparation	Age (years) (N)	Hypogonadism	GH	Design	Outcomes	Results	Comments
2019	(42)	E2	E2	13-20 (N=40)	Yes	off at least for 6 months	Randomized clinical trial	Genotoxic estrogen#	E2: oral > TD	Another evidence for E2-TD
					Estrogen off at least for 6 m		(probably a sub study of ref. 43)			
2013	(43)	E2	E2	13-20 (N=40)	Yes	off at least for 6 months	Randomized clinical trial	Body composition, Bone accrual	ND (not different)	The most extensive study so far designed
		1, 1.5, or 2.0 mg with dose-titration	0.0375~0.1 mg with dose-titration		Estrogen off at least for 6 m		E2 was titrated on LC-MS/MS control levels	Lipid oxidation, Resting energy expenditure	ND	
								Lipids, glucose, osteocalcin Highly sensitive CRPSuppression of Gn	ND	
								IGF-I	oral < TD	
								E2 metabolites such as E1	oral > TD	
2009	(44)	CE	E2	10 or older (N=12)	Prepubertal	Treated	Randomized clinical trial	Bone accrual, Uterine growth	E2-TD > CE-oral	GH was also injected
		0.3 mg for 6 m, 0.3/0.625 mg for another 6 m (daily dose)	0.025 mg for 6 m, 0.0375 mg for another 6 m (twice weekly)					Pubertal development, Plasma Lipids, IGF-I	ND	
2007	(45)	E2 for 6 wk	E2 for 6 wk	11-15 (N=11)	Prepubertal	Treated	Prospective, cross-over with a 4-wk washout	Protein Turnover, Lipolysis Lipid oxidation rate	ND	GH was also injected
		0.5, 1.0, 2.0 mg for 2 wk (daily dose)	0.025, 0.0375, 0.05 mg for 2wk (twice weekly)					Plasma Lipids, Insulin Fibrinogen, IGF-I		

#Genotoxic estrogen are estrogen metabolites which are known to be linked to breast carcinogenesis in post-menopausal women.

E2, 17β estradiol; EE2, ethinyl estradiol; CE, conjugated estrogen; MPA, medroxyprogesterone acetate; TD, transdermal; Gn, gonadtropins.

Oral estrogen administration is thought to be associated with two, potentially deleterious long-term effects. One is the increased risk of cardiovascular outcomes. Although this risk increases after menopause in the general population (46, 47), whether patients with TS also have an increased risk is unclear. The other, potentially deleterious effect is the higher level of genotoxic estrogens in adolescent TS patients (at the age of 13-20 years) receiving oral E2 instead of transdermal E2 (42), which may lead to an increased risk of estrogen-dependent malignancies, such as some forms of breast cancer. However, there are as of yet no epidemiological data demonstrating an actual link between genotoxic estrogens and cancer. Indeed, in their epidemiological study of well over 1000 patients with TS, Viuff et al. demonstrated that the risk of breast cancer was actually lower in TS, even during hormone replacement therapy (48).

There are pressing reasons not to wait long before initiating estrogen therapy, although pubertal induction may limit the ability to achieve adult height. The major, clinical targets of estrogen replacement therapy also should be considered. Breast development has been a focus of research in TS for over a decade (13, 49). For physical as well as psychosocial reasons, healthy breast development is desirable. A recent study of this topic (50) found that the results of breast development in TS were not adequate even with intervention. Furthermore, some patients respond poorly to treatment in practice. The poor responders may have severe webbed neck and lymphoedema or they may be clinically emaciated.

Although uterine growth is another target of therapy, only a few studies on this topic have been published (44, 49, 51). Recently, researchers used a three-dimensional approach, which is more accurate than a two-dimensional approach, to demonstrate that the recommended induction method increased uterine volume significantly. However, the uterine volume was still smaller than the value in age-matched, healthy teenagers and adults (52).

Another critical effect of estrogen replacement therapy relates to bone health (53). Epidemiological studies have reported an increased prevalence of bone fractures in TS (54–56), although some controversy still surrounds this issue (57, 58). Decreased bone mineral density is considered one of the causes of the fractures in TS. Estrogen replacement increases BMD (59), making early induction essential to bone mineral accrual (57, 59–61). More advanced technologies, such as peripheral quantitative computed tomography, are needed to assess bone density and geometry (51, 62).

Other significant effects that may be likely related to pubertal induction are psychosocial outcomes and cardiovascular health. The impact of induction therapy on psychosocial wellbeing is not negligible since the period during which induction is performed is when children typically begin to acquire their sense of autonomy (reviewed in 29) and coincides with the period of physical development in patients with TS. Another potential reason for timely pubertal induction in patients with TS is the implications for neurocognitive development (63, 64) although this topic is beyond the scope of the present monograph.

Finally, epidemiological data suggest that estrogen is related to cardiovascular heath in the long run, as extensively illustrated in the most recent and widely recognized guideline (2). However, to the best of our knowledge, the impact of estrogen during pubertal induction remains unknown as discussed later.

#### Progestin

Progestin must be used in addition to estrogen when breakthrough bleeding occurs or after 2-3 years of pubertal induction with estrogen (1–19, 65). However, recent two guidelines emphasized that progestin should be added after confirming uterine size and endometrial thickness on ultrasonography (5, 7).

Adding progestin reduces the risk of endometrial cancer associated with unopposed estrogen (66, 67) and minimizes irregular bleeding and endometrial hyperplasia (3). The many formulations currently available have been summarized by Klein et al. (3, 7). Micronized progesterone or dydrogesterone (3, 7, 65) is recommended.

When beginning pubertal induction in patients with TS, cyclic progestin administration, typically for 12–14 days once a month or in a cycle, is the preferred method. Pubertal induction should induce menstruation as this is important for the patients’ mental and emotional health. Furthermore, because cyclic therapy promotes normal cyclical endometrial repair, it is more beneficial in patients attempting to conceive through oocyte donation (65).

Far less research has been conducted on progestin therapy than on estrogen therapy. For example, although most guidelines for TS recommend oral progestin, they rarely discuss other types of progestin administration, such as vaginal administration, which may mimic the physiological uterine tissue concentration more closely.

#### Estrogen and progestin combination therapy, including oral contraceptives (OCs)

Several types of patches and pills are available for estrogen and progestin combination replacement therapy in addition to OC pills. A recent review has listed these products as being available worldwide (3, 7). OC pills are used in North America and Europe by healthy, young girls. As a result, late-adolescent patients with TS after menarche may want to use these products to feel more like their peers. However, the long-term consequences of using these products, such as their effects on cardiovascular and bone health, still need to be clarified. Furthermore, it is worth noting that most of these products are not recommended for the initial phase of pubertal induction (2, 3).

### Current recommendations for ERT for pubertal induction (Current E2-TD therapy)

The most recent and extensive guideline for TS, published in 2017 (2), recommends that ‘[low-dose, transdermal estradiol therapy]’ be initiated between age 11 and 12 years in patients with TS [without pubertal development]. If arrested pubertal development is predicted, the guidelines recommend increasing [to the adult dosage] for two to three years. Adding progestin is also advised once breakthrough bleeding occurs or after two years of estrogen treatment. These recommendations are mainly for patients with poor endogenous estrogen production, and the guideline offers no concrete methods of performing [‘low-dose transdermal estradiol therapy’]. For example, they do not explain how to calibrate the dosage during induction precisely. However, a concrete method of increasing the induction dosage using transdermal E2 is described in a follow-up study (3).

Recently, a group of experts with the European Society for Pediatric Endocrinology published a physiological method of pubertal induction using transdermal estradiol therapy in patients with TS without endogenous estrogen production (7). The recommended age for initiating this therapy is 11 to 12 years, and the recommended initial dosage is 1/16-1/24 of the adult dosage, mainly to be administered at night to mimic the nighttime elevation of physiological estradiol (7). The dosage and rationale for mimicking the normal physiological E2 serum concentration was explained in detail in previous studies (68, 69). While mimicking the nighttime E2 levels results in a close approximation of the physiological serum estradiol concentration profile, the consent of the patient’s’ parents is required before it is used, and their cooperation in encouraging adherence is required during the treatment course.

The previously cited recommendations (2, 3, 7) are currently widely accepted as a proposed form of induction therapy for TS (abbreviated as “current E2-TD therapy” in this monograph) (**Table 4**). **Table 4** shows the advantages and disadvantages of this therapy in comparison to other therapies.

**Table 4:** Advantages and disadvantages of EE2-O therapy, modified EE2-O therapy, current E2-TD therapy, and ideal E2-TD therapy.

Full explanation	Ref.	Initiation	Initial dosage	Advantages	Disadvantages
EE2-oral therapy #	(70, 71)	5-9 yrs	25 ng/kg/day	First, seminal work, RCT	Not recommended, too early starting age (as early as 5 yrs)
				Mimicking physiology	Use of oral EE2 ##
Modified EE2-oral therapy	(74)	9-13	1-5 ng/kg/day	Initiated lower than EE2-oral or Current E2-TD therapy	BMD accrual is suboptimal
				Good final/adult height data	Use of oral EE2 ##
Current E2-TD therapy	(2–14)	11-12	3-10% of AD	Mostly accepted as appropriate	Scareced long-term evidence
				Recommended by many societies	Patches are not available in some countries
				Final/adult data is promising	Some patches cannot be cut smaller
					Uterine growth may be suboptimal
Ideal E2-TD therapy ### (suggested here)		9	~1% of AD	Initiated lower than in current E2-TD therapy	Completely new Tx (not evidenced)
				Can be started at earlier ages	No final/adult height data is available
				More physiological initiation	No short-term or long-term data is available

# One of the recent reviews (Gravhold et al. in 2017) referred to this therapy as 'very-low-dose estrogen supplementation'.

## The oral route is not physiological due to the first-pass effect while the transdermal route is more physiological in terms of absorption into the circulation.

Further, EE2 is a synthetic estrogen whereas E2 is the most potent, physiological form of estrogen.

### Ideal E2-TD therapy is characterized by earlier, lower-dosed initiation.

E2, 17β estradiol; EE2, ethinyl estradiol; CE, conjugated estrogen; MPA, medroxyprogesterone acetate.

E2-TD, E2 transdermal.

AD, adult dose.

Few studies have examined adult height or bone mineral density accrual in TS patients receiving current E2-TD therapy. A recent study found that uterine development in women with TS receiving current E2-TD therapy (52) was suboptimal compared with that of healthy women.

## Our suggestions for ideal pubertal induction

### Our initial study: modified EE_2_-O therapy 

The benefits of low-dose estrogen therapy for women with TS were initially discussed in 2011 by Ross et al. (70) and in 2014 by Quigley et al. (71). These seminal studies examined the effects of oral ethinyl estradiol (EE2) starting at 25 ng/kg/day; the adult dosage for complete hypogonadism is 100–200 ng/kg/day. The studies attempted to mimic the physiological increase in estrogen by starting treatment at a low dosage, then gradually increasing it with maturation in a form of treatment, described as EE2 oral (EE2-O) therapy (**Table 4**).

Although the concept of EE2-O therapy is physiologically sound, there are some concerns. First, the therapy reportedly begins as early as age 5 years although the 95% CI for the onset of breast development in the US ranges from 8.8 to 10.3 years (20). The study was also designed to analyze the impact of this therapy on neurocognition in TS (72, 73). Second, the initial EE2 dosage of 25 ng/kg is not particularly low, in comparison to the adult dosage of this compound. Indeed, some of the subjects in this study (the exact proportion was not described) showed breast development at this dosage (70). EE2-O therapy is not recommended in the most recent guideline (2), which state, “We suggest not routinely [adding] very-low-dose estrogen supplementation in the prepubertal years to further promote growth.” The observation regarding “very-low-dose estrogen supplementation” in ref. 2 implicates the historical studies using EE2 discussed above (70, 71), although this is not explicitly stated in the reference. Our previous work (74) using an even lower EE2 dosage for induction is not mentioned in the guideline in question (2).

We recently published a study examining the effects of induction using lower-dose estradiol therapy (74) (modified EE2-O therapy in **Table 4**), which begins with an oral dosage of 1–5 (mostly 1–2) ng/kg/day. The lower starting dosage was assumed to approximate more closely the increase in physiological estrogen. Our study demonstrated that the initial EE2 dosage did not induce breast development and recorded final/adult height (height velocity < 2 cm/year) of 152.4 +/- 3.4 cm (+2.02 +/- 0.62 SD for TS) (N=17), which was significantly greater than that of a control group who received a different treatment (148.5 +/- 3.0 cm; +1.30 +/- 0.55 SD). The mean, female, Japanese, adult height was reportedly about 158 cm with no secular trend (75, 76). Our data on adult height in the study, which are among those of the highest quality published thus far (1), suggested that the modified EE2-O therapy did not compromise height acquisition despite early initiation. The findings of our first study (74) led to the formation of the ideal, estradiol, transdermal therapy (ideal E2-TD therapy) explained below.

In hindsight, there are a number of issues related to our study. EE2 is a synthetic estrogen whereas estradiol is the most potent physiological form of estrogen. Owing to the first-pass effect (**Table 4**), the oral route cannot replicate the natural secretion of estrogen. Additionally, starting estrogen therapy earlier and increasing the dosage more slowly than in our study may be necessary from a physiological point of view (see below) and would likely have led to a better BMD outcome than observed in our initial study (74).

### Background knowledge and concept of ideal E2-TD therapy with earlier, lower-dose transdermal E2 administration

This section discusses the rationale for ideal E2-TD therapy in greater detail than our previous publications on the topic [(77, 78) as an erratum of 79]. The rationale of pubertal induction for TS without endogenous ovarian function includes the following:

- Use of E2 as a physiological form of estrogen
- Use of the transdermal route
- Gradual dose escalation with age as consistent with serum E2 concentration (physiological age at the induction)
- Introduction of menarche at the appropriate age

To further improve outcomes observed in our modified EE2-O therapy, a couple of issues should be considered. First, the recent guidelines clearly recommend transdermal E2 administration (2–12). Oral estrogens are reportedly associated with an increased risk of poor cardiovascular outcomes in the general, post-menopausal population although there is some controversy about the issue (46, 47, 79, 80) and this finding has not yet been established in patients with TS. It is obvious that transdermal administration is more physiological as discussed above.

Furthermore, earlier, lower-dose estrogen therapy approximates more closely to physiology, which may lead to improved height prognosis and bone mineral acquisition. As shown, final/adult height and BMD data were still suboptimal in our previous study (74). Moreover, ideal pubertal induction might achieve more appropriate uterine growth. Even in TS patients with the current E2-TD therapy, the uterine growth was less than ideal (52).

There are fundamental observations necessary for the understanding of ideal pubertal induction. First, breast development seems to be related to the amount of estrogen exposure over time, and the initial release of estrogen probably starts before breast development. Indeed, on a longitudinal growth velocity curve, the annual growth rate (cm/year) at each age reaches a nadir before the onset of breast development. This minimum value and the ensuing increase in the annual growth rate are referred to as ‘nadir’ in the present review. This change in growth velocity may reflect earlier estrogen production before breast 2 Tanner stage; estrogen is sufficient for growth acceleration but not for breast development. Tanner et al. reported the same phenomenon in their landmark study (81).

**Figure 1:**
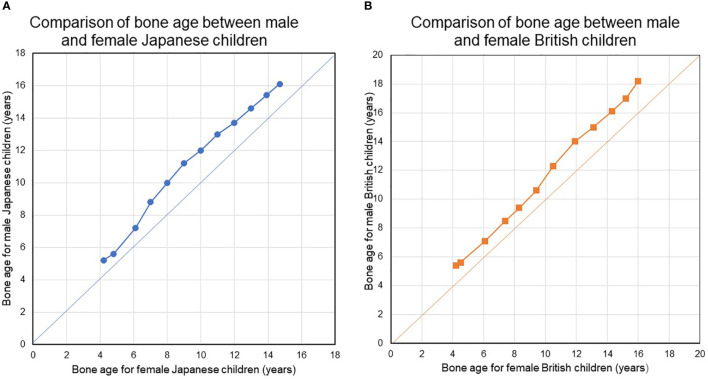
**(A, B)** Compare bone ages between females and males in Japanese and British children. During childhood and adolescence, bone maturation advances faster in girls than in boys at all ages. During childhood and adolescence, bone maturation advances faster in girls than in boys at all ages. BA was evaluated using the original TW2-Radius-Ulna-Short bones (RUS) method and the TW2-RUS method standardized for Japanese children, for British children, and for Japanese children, respectively.

Second, bone maturation during childhood as assessed in terms of bone age occurs earlier in female. **Figure 1** show the results of an analysis of bone age (BA) with sex-related differences in bone maturation. The analysis in **Figure 1** used a bone age atlas for female Japanese children (82) as a reference. When radiographs in the atlas for female, Japanese were evaluated using a scoring system designed for male, Japanese children, the latter demonstrated a BA increase at all ages, suggesting that bones develop more quickly in females (**Figure 1**). The radiographs in the atlas for female, Japanese children were also evaluated using a scoring system designed for female and male British children based on the TW2-Radius Ulna Short bones (RUS) method (82, 83). Faster bone maturation was also observed in the female, British subjects (**Figure 1**).

BA is a surrogate marker of bone maturation and is at least partially dependent on estrogen function in both sexes, as exemplified by BA retardation in a 46,XY patient with estrogen receptor α defect (84) and in male and female patients with aromatase deficiency (85, 86). Estradiol levels may gradually increase before breast development. Indeed, bioassays have found a higher serum estradiol level in healthy, prepubertal female subjects as young as 4-5 years than in male subjects (87, 88). This early, slow rise in estradiol suggests that ideal E2-TD therapy may best be started before the average age at the nadir and the start of breast development. **Table 5** shows the estimated serum E2 concentration for each pre-pubertal or pubertal stage. Pubertal induction therapy in TS without gonadal function ideally mimic the E2 levels shown in **Table 5** *via* earlier, lower-dose, transdermal E2 administration.

**Table 5:** Estimated serum estradiol levels and estrogen effects in female children.

Estrogen effect	Age (yrs)	E2 in standard immunoassay	E2 in bioassay	Ref.
Bone maturation	> ~4	Not detectable (ND)	female > male	see **Figure 1**
Initial increase in growth velocity	9.5	ND	female > male	(83)
Breast development	10	ND initially		(20)
Menarche/Uterine maturation	12.5	Detectable		(22)
		Less than adult levels		
		Female > male		
(Adult female subjects)		~100 pg/ml (depending on the timing of the cycle)		

E2, 17β estradiol.

Theoretically, ideal pubertal induction therapy has the following targets:

- Appropriate final/adult height, not compromised by estrogen replacement
- Appropriate quality of life for pubertal female, including the achievement of healthy pubertal development
- Appropriate acquisition of bone mineral density at final/adult height

Of course, ideal E2-TD therapy suggested here have never addressed any of these points.

### Our practical proposal for an ideal induction therapy: Suggestions for a more physiological approach

**Tables 6**, **7** shows our proposal for the clinical application of ‘ideal E2-TD therapy’ with earlier, lower-dose E2 initiation, together with current, widely accepted estrogen therapy (current E2-TD therapy, see also **Table 4**). **Table 6** shows the initial dosage required for pubertal induction (89) and **Table 7** shows the precise method of calibrating the increases in estrogen dosage.

**Table 6:** Comparisons between current and ideal# pubertal induction using estrogen in TS without endogenous estrogen.

	Preparations	Puberty induction [Adult] dose	Initiation age (year)
Current (modified from ref. 2, 89)			
		~3 to 10% of [adult dose]	11~12

	E2-TD	3-7 [75-150] mcg/day##	
	E2-oral	0.25 [1-2] mg/day	
	EE2-oral	2 [10-20] mcg/day	
	CE-oral	0.0625 [0.625-1.25] mg/day	
Ideal (earlier, lower-dose estrogen initiation)#			
		< 1% of [adult dose]	9

	E2-TD	~1 [75-150] mcg/day##	
	E2-oral	~0.01 [1-2] mg/day	
	EE2-oral	~0.1 [10-20] mcg/day	
	CE-oral	~0.00625 [0.625] mg/day	

# This ideal therapy is suggested here. ## The dose is variable for each patch.

Lower-dose estrogen may not be available in some of patches or tablets.

Mean age of breast development is assumed to be at the age of 10 years in this table.

E2, 17β estradiol; EE2, ethinyl estradiol; CE, conjugated estrogen; MPA, medroxyprogesterone acetate.

E2-TD, E2 transdermal; ERT, estrogen replacement therapy.

**Table 7:** Examples of dose escalation of Current and Ideal (earlier, lower-dosed)#, estrogen pubertal induction using estrogen in TS without endogenous estrogen.

	TD	TD	TD	Transderm (TD)	TD	Oral	Oral	Oral
**Preparation**	E2	E2	E2	E2	E2	E2	E2	CE or EE2*
**Current/Ideal (ref)**	Current	Current	Current	Ideal#	Ideal#	Current	Ideal#	Ideal#
	(ref. 3)	(ref. 7)	(ref. 4)	(early)	(late)	(ref. 4)		
**Induction age**	11~12 yrs	11~12	11~12	9	9	12	9~10	9~10
**Examples of patch/tab**	25~100 mcg	25, 50 mcg	25~100 mcg	0.09~0.72 mg/2 days	0.09~0.72 mg/ 2 days	0.25~2 mg	0.25~2 mg	0.625 mg or 2 mcg
**Adult dose (AD)**	variable	50-75 mcg/day	100 mcg/wk	0.72 mg/2 days	0.72 mg/ 2 days	2 mg daily	2 mg daily	0.625 mg or 10 mcg daily
**Age (yrs)**	**Ratio of adult dose (RAD)**	**RAD**	**RAD**	**RAD**	**RAD**	**RAD**	**RAD**	**RAD**
9	0	0	0	~1/120	~1/120	0	0	1/128
				(1/8 of 0.09 mg patch for 2 days x 2/wk)				
10	0	0	0	1/64	1/64	0	1/56	1/64
				(1/8 of 0.09 mg patch for 2 days)			(1 day/wk)	
11	5-10%	1/16	1/32	1/32	1/32	0	3/56	1/32
				(1/4 of 0.09 mg patch for 2 days)			(3 days/wk)	
11.5	25%	1/16	1/16	1/16	1/32	0	5/56	1/16
				(1/2 of 0.09 mg patch for 2 days)			(5 days/wk)	
12	50%	1/8	1/8	1/8	1/16	1/8	1/8	1/8
				(0.09 mg patch for 2 days)				
12.5	75%	1/8	3/8	1/4	1/16	1/4	1/4	1/4
				(0.18 mg patch for 2 days)				
13	100% (AD)	1/3	1/2	1/2	1/8	1/2	1/2	1/2
*** ***				(0.36 mg patch for 2 days)				
13.5		1/3	3/4	100% (AD)	1/8	3/4	3/4	3/4
14		100% (AD) *** *** *** ***	100% (AD) *** ***		1/4	100% (AD)	100% (AD)	100% (AD)
14.5					1/2			
15					100% (AD)			

E2, 17β estradiol; EE2, ethinyl estradiol; CE, conjugated estrogen.

AD, adult dose.

First, it should be borne in mind that the therapy proposed here as ‘ideal’ is a preliminary version and is not based as of yet on any evidence. The idea awaits testing in a future study. In **Tables 6** and **7**, breast development is assumed to start roughly at age 10 years world-wide for the sake of the simplicity (20). The initial dosage at age 9 years is roughly 1/100th of the adult dosage. Not only the transdermal E2 regimen but also the oral EE2 and CE regimens are shown, because transdermal E2 patch is still unavailable in some regions (15, 16). There are two protocols using transdermal E2 patch for patients aged 9 years or older, either of which can be used depending upon patients’ psychological age, height, BA, BMD, and the consent of the patients and their guardians (**Table 7**).

Several clinical considerations must be made before administrating this ideal E2-TD therapy in patients with TS. First, if patients do not have a high FSH level (> 10 mIU/mL at age10 years or older, > 6.7 mIU/mL at age 6 to 9 years) (24, 35), treatment should await confirmation of the absence of pubertal development, or an increase in FSH. The FSH level (as discussed above) is particularly helpful in regions where AMH cannot be measured. Second, treatment initiation depends on patients’ racial background; for instance, patients of African descent mature earlier than their European counterparts (20).

Last but not least, initiation of estrogen treatment at age 9 years as shown in **Tables 6**, **7**, is tentative. Given the size of E2 patches, the minimum dosage shown in those Tables is the lowest possible dosage available for each formulation. If a smaller transdermal estradiol patch were used, the ideal age at ERT initiation in patients with TS without endogenous estrogen production might be around 5 years, as discussed above (87, 88).

### Prospects

Undoubtedly, further research is needed to gather evidence to determine whether the ideal E2-TD therapy is viable. First, the final/adult height data obtained with the ideal E2-TD therapy are unclear. Our first study of modified EE2-O therapy produced some of the best adult height outcomes (final/adult height; roughly -1.1 SD for reference Japanese female adult) (1, 74). Our recent experience in outpatient clinic, the outlook for the ideal E2-TD therapy is promising.

Second, QOL data, including satisfaction with breast size (50), should be evaluated in patients receiving the ideal regimen. The age of menarche in **Table 7** is 13 to 14 years, which does not represent a significant delay, suggesting that the deterioration in QOL may not be caused by the delay in menarche.

Third, further research is needed to improve BMD outcomes. The BMD values obtained in our initial study of modified EE2-O therapy (74) were suboptimal as discussed. Our research team (90) and others (91) have published further, prepubertal data on TS indicating that BMD begins to decrease at around age 7–10 years. Therefore, future studies of therapeutic approaches should aim at achieving better BMD outcomes. Similarly, uterine volume in patients treated with the current E2-TD therapy was also suboptimal as above explained (52). To attain better BMD and uterine development, estrogen therapy may be started earlier in patients with TS than indicated in **Table 6** or **7**. Furthermore, LC-MS/MS may be preferable for monitoring the serum E2 level in patients receiving earlier, lower-dose, transdermal E2 because it has a much lower sensitivity than immunoassays. Monitoring the E2 level and calibrating the dosage using LC-MS/MS measurements were done in one of the most elegant studies to date (42, 43).

Of course, a balance needs to be struck in practical medicine between the scientific ideal and the realities of clinical practice. For example, an RCT comparing two treatment plans through pubertal induction to adulthood is not feasible or patient-oriented; on the other hand, a single-arm trial using transdermal E2 may be practical for assessing the quality of the long-term prognosis. An international, collaborative study would be ideal for reaching a consensus on clinically relevant matters.

## Author contributions

YH planned the review, searched for previous articles, and wrote the first draft of the manuscript. MS analyzed the bone age data. All authors contributed to the article and approved the submitted version.
